# Postoperative survival effect of the number of examined lymph nodes on esophageal squamous cell carcinoma with pathological stage T1–3N0M0

**DOI:** 10.1186/s12885-022-09207-x

**Published:** 2022-01-28

**Authors:** Lei-Lei Wu, Jiu-Di Zhong, Jia-Li Zhu, Lu Kang, Yang-Yu Huang, Peng Lin, Hao Long, Lan-Jun Zhang, Qi-Long Ma, Li-Hong Qiu, Guo-Wei Ma

**Affiliations:** 1 Sun Yat-sen University Cancer Center, State Key Laboratory of Oncology in South China, Collaborative Innovation Center for Cancer Medicine, Sun Yat-sen University, Guangzhou, 510060 People’s Republic of China; 2 Department of Thoracic Surgery, Shanghai Pulmonary Hospital, School of Medicine, Tongji University, Shanghai, People’s Republic of China; 3 Guangdong Esophageal Cancer Institute, Guangzhou, 510060 People’s Republic of China; 4 School of Medicine, Tongji University, Shanghai, People’s Republic of China; 5 Jinling Hospital, Nanjing, 210000 People’s Republic of China; 6 The Department of Thoracic Surgery, Sun Yat-sen University Cancer Center, 651 Dongfengdong Road, Guangzhou, 510060 People’s Republic of China

**Keywords:** Esophageal squamous cell carcinoma, Overall survival, Stage T1–3N0M0, Lymph node, SEER

## Abstract

**Background:**

The postoperative survival effect of the number of examined lymph nodes on patients of R0-resected esophageal squamous cell carcinoma with pathological stage T1–3N0M0 is still unclear.

**Methods:**

Patients diagnosed with pathological stage T1–3N0M0 esophageal squamous cell carcinoma from two cancer databases—our cancer center (*N* = 707), and Surveillance Epidemiology and End Results (*N* = 151). The primary clinical endpoint was overall survival. The X-tile software was used to determine the optimal cutoff value of the number of examined lymph nodes, and propensity score matching was conducted to reduce selection bias according to the results of X-tile software. The cohort of 151 patients from another database was used for validation.

**Results:**

X-tile software provided an optimal cutoff value of 15 examined lymph nodes based on 707 patients, and 231 pairs of matched patients were included. In the unmatched cohort, Cox proportional hazard regression analysis revealed better overall survival in patients with more than 15 examined lymph nodes (adjusted hazard ratio, 0.566, 95% confidence interval, 0.445–0.720; *p* < 0.001) compared with patients with 15 or fewer examined lymph nodes. In the validation cohort, patients with more than 15 examined lymph nodes also had better overall survival (adjusted hazard ratio 0.665, *p* = 0.047).

**Conclusions:**

The number of examined lymph nodes is a significant prognostic factor in esophageal squamous cell carcinoma patients with pathological stage T1–3N0M0, and more than 15 examined lymph nodes are associated with better overall survival. Although the difference is not significant, the survival curve of patients with examined lymph nodes > 30 is better than those with examined lymph nodes 15–30. We believe that the number of examined lymph nodes can provide prognostic guidance for those patients, and the more examined lymph nodes cause lesser occult lymph nodes metastasis and lead to a better prognosis. Therefore, surgeons and pathologists should try to examine as many lymph nodes as possible to evaluate the pathological stage precisely. However, we need more validation from other studies.

## Background

In the global cancer spectrum, the incidence and mortality of esophageal carcinoma rank 9th and 6th, respectively [1]. Esophageal squamous cell carcinoma (ESCC) is the major histological subtypes of esophageal carcinoma [1, 2]. More than half of the newly diagnosed esophageal carcinoma cases occur in China, importantly, ESCC accounts for more than 90% of esophageal carcinoma cases [1, 3]. The postoperative prognosis for ESCC patients remains poor [4]. The previous research had identified specific factors that have a direct influence on prognosis of esophageal carcinoma. Some studies had focused on the survival impact of the number of examined lymph nodes (NELNs) on esophageal carcinoma patients [5–11]. However, above studies provided differently optimal NELNs for esophageal carcinoma patients. Besides, the optimal number of lymph nodes given in the existing guidelines is also variable. National Comprehensive Cancer Network recommends at least 15 lymph nodes to be removed in the operation, while The American Society of Clinical Oncology recommends surgeons to perform adequate nodal dissection with at least 16 to 18 lymph nodes, preferably more than 20 [12, 13]. For R0-resected ESCC patients with stage T1–3N0M0, the prognostic significance of NELNs remains unclear, and survival outcomes remain to be heterogeneous and difficult to estimate [8, 10]. Existing guidelines from the National Comprehensive Cancer Network and National Health Commission of the People’s Republic of China don’t recommend that R0-resected ESCC patients of stage T1–3N0M0 need to receive adjuvant therapy [12, 14], however, for those patients with inadequate nodal dissection, adjuvant therapy may be needed because of their poor prognoses.

Therefore, determining the appropriate number of examined lymph nodes for patients with pathological stage T1–3N0M0 treated with R0 resection would help clinicians in identifying those with poor prognosis and promote more accurate follow-up recommendations and adjuvant treatment (such as radiotherapy). Thus, this study aimed to obtain an appropriate number of examined lymph nodes with improved prediction of long-term survival in this patient population.

## Patients and methods

### Patients

**Fig. 1:**
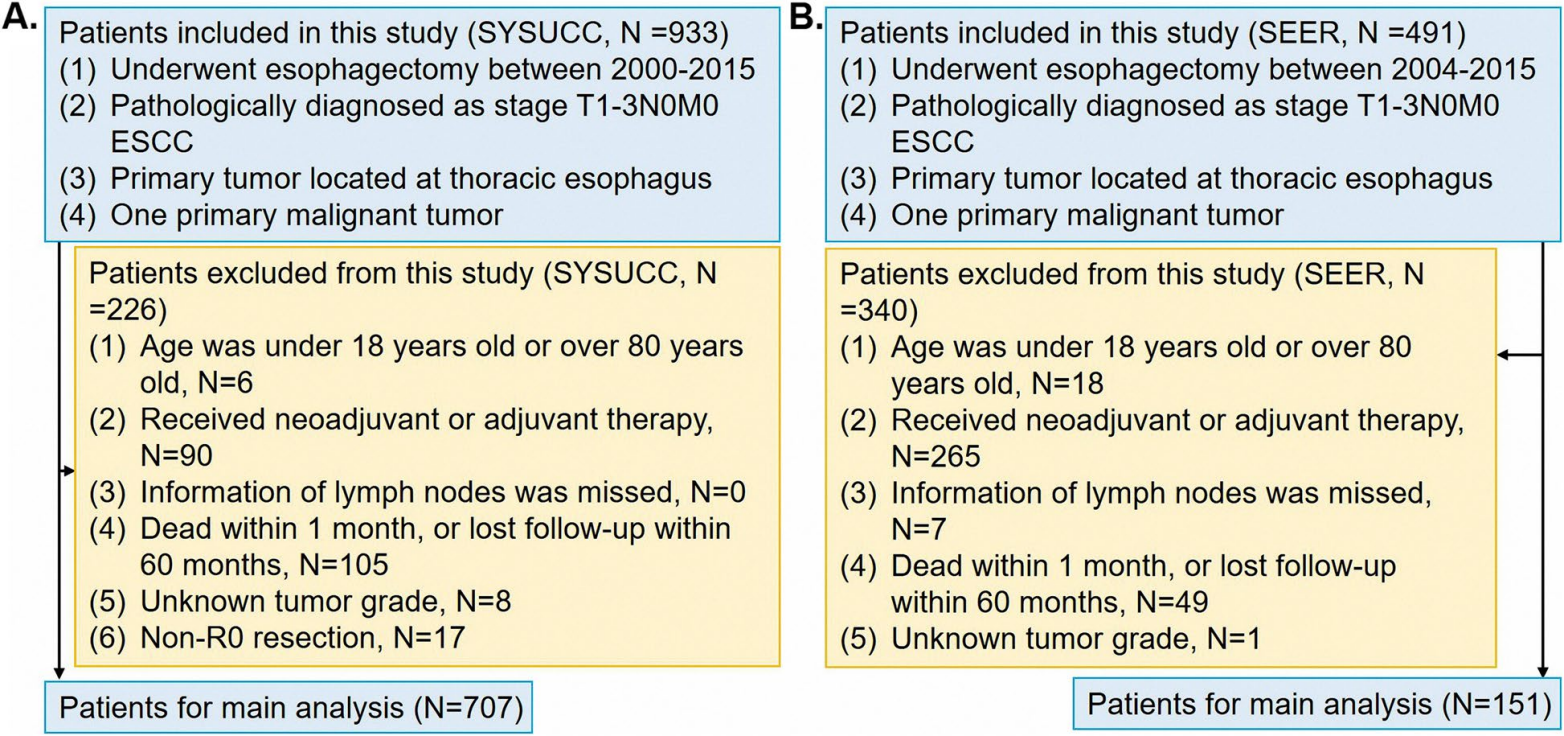
The diagram of the patient screening process in the SYSUCC and SEER database (SYSUCC Sun Yat-sen University Cancer Center, SEER Surveillance Epidemiology and End Results, AJCC American Joint Committee on Cancer Staging Manual)

The study was approved by the Clinical Research Ethic Committee of Sun Yat-sen University Cancer Center (approval number: YB2016–072), and informed consent of patients was waived. A total number of 707 patients who underwent esophagectomy at the Department of Thoracic Surgery of our cancer center between 2000 and 2015 were enrolled retrospectively in the present study. These patients underwent neck-abdomen computed tomography scans, barium esophagography and the esophagus endoscopy before the operation. Surgeons evaluated the status of celiac lymph nodes by neck-abdomen computed tomography scans. Patients eligible for this cohort study had pathologically confirmed pathological stage T1–3N0M0 according to the 8th edition of American Joint Committee on Cancer Staging Manual. We confirmed the pathological stage according to the histological records and reports of computed tomography scans. The flow chart of the study is shown in Fig. 1. Besides, the data obtained from SEER database were conducted to validate the findings from our cancer center. According to the similar screening criteria, there were 151 patients selected from SEER database as validation cohort. These patients underwent operation between 2004 and 2015. The surgical procedure consisted of Sweet, Ivor-Lewis, or McKeown esophagectomy, determined by the location of the tumor, extent of the disease, and surgeon’s preference.

### Follow-up

At our cancer center, those patients were regularly followed up by telephone from professional follow-up department. The median follow-up time from surgery to the last contact with patients was 80.0 months (range 2–192 months). The final follow-up date was 3rd September 2019, and all 707 patients were observed. We recommended that the patients came to the outpatient department for a follow-up examination every 3–6 months for the first 2 years, then every 6 months for the next 3 years, and then every year after that. Follow-up examinations consisted of history assessment, barium esophagography, physical examination, chest radiography, cervical ultrasonography, abdominal ultrasonography, and neck-abdomen computed tomography scans. If necessary, patients underwent positron emission tomography- computed-tomography, endoscopy, or both. In the SEER database, the median follow-up time was 34 months (range 1–155 months). Given overall survival was most clinically relevant, we considered overall survival as a primary clinical endpoint.

### Statistical analysis

Statistical analysis was performed using SPSS Statistics 25.0 software (IBM SPSS, Inc., Chicago, IL, USA), and X-tile version 3.6.1 (http://www.tissuearray.org/rimmlab). Chi-squared statistical test, Fisher exact test and Mann-Whitney *U* test were used to determine the association between clinical information and groups. Before Mann-Whitney *U* test, we used Shapiro-Wilk test and Kolmogorov-Smirnor test to validate the non-normal distribution of continuous data. Interquartile range was used to access the dispersion of data. We used X-tile software to determine the optimal cutoff value of NELNs. Previous studies had revealed that X-tile software was similar to time-dependent Receiver Operating Characteristic curve analysis, and could provide an optimal cutoff value for continuous data [10, 11, 15–17]. Hazard ratios (HR) with 95% confidence intervals (95% CIs) were calculated by univariable and multivariable Cox proportional hazard regression analyses. Univariable analysis was used to evaluate the effect of clinicopathological factors on overall survival. Multivariable analysis was performed to identify the NELNs as an independent prognostic indicator after adjusting for other factors. Variables with univariable analysis had *p* < 0.05 or affecting prognosis (such as sex, age, two-field dissection and three-field dissection) were selected to enter in the multivariable analysis. In addition, Kaplan–Meier analysis and the log-rank tests were used to compare survival curves between different groups. It was considered statistically significant that the results of all statistic test met a two-sided *p* values of *p* < 0.05.

For the cohort from our cancer center, propensity score matching was conducted to reduce selection bias, and to make the results of analyses more reliable [18, 19]. Propensity scores were estimated using a logistic regression model based on covariables including age, sex, primary tumor location, tumor differentiation, surgical approaches, and pathological tumor (pT) stage that might affect survival. One-to-one matching without replacement was employed with a caliper width of 0.01. Besides, the random number seed was 123,456. The quality of matching was evaluated by comparison of each covariable after propensity score matching. Patients from SEER database was discreted into two subgroups using the same cutoff value of NELNs defined in the data of our cancer center.

## Results

### Patient characteristics

**Fig. 2:**
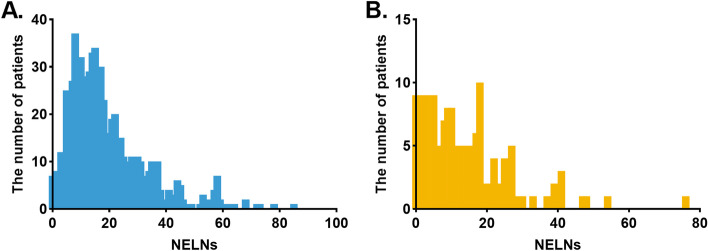
The distribution of lymph-node count in Sun Yat-sen University Cancer Center (**A**) and Surveillance Epidemiology and End Results database (**B**)

The clinical characteristics of the patients from Sun Yat-sen University Cancer Center are listed in Table 1. Among the 707 patients, 515 (72.8%) patients were men and 192 (27.2%) were women. The patients’ age ranged between 28 and 79 years (median, 59 years). In this cohort, the 1-, 3- and 5-year overall survival rates were 89.0, 71.0, and 62.0%, respectively. In the SEER cohort, the 1-, 3- and 5-year overall survival rates were 56.0% vs. 42.0% vs. 19.0%, respectively, and the median survival time was 34.0 months. The clinical characteristics of the patients in the SEER are listed in Table 2**.** The median NELNs was 16.0 ± 13.6 and 10.0 ± 12.3 in single cancer center and SEER databases, respectively. The distribution status of NELNs from SEER and our cancer center is presented with Fig. 2. We used X-tile software to determine the optimal cutoff value of NELNs as 15 based on data of cancer center.

**Table 1:** Clinicopathological characteristic of patients from Sun Yat-sen University Cancer Center database before and after propensity score matching

	Entire cohort (*N* = 707)			Matched cohort (*N* = 462)		
	NELNs ≤15 (*N* = 352)	NELNs > 15 (*N* = 355)		NELNs ≤15 (*N* = 231)	NELNs > 15 (N = 231)	
Variables			*P* value			*P* value
Sex, n (%)			0.360^*^			0.212^*^
Male	251 (71.3%)	264 (74.4%)		173 (74.9%)	161 (69.7%)	
Female	101 (28.7%)	91 (25.6%)		58 (25.1%)	70 (30.3%)	
Drinking history, n (%)			0.001^*^			0.761^*^
No	271 (77.0%)	235 (66.2%)		163 (70.6%)	160 (69.3%)	
Yes	81 (23.0%)	120 (33.8%)		68 (29.4%)	71 (30.7%)	
Smoking history, n (%)			0.004^*^			0.774^*^
No	152 (43.2%)	116 (32.7%)		87 (37.7%)	90 (39.0%)	
Yes	200 (56.8%)	239 (67.3%)		144 (62.3%)	141 (61.0%)	
Tumor differentiation, n (%)			0.373^*^			0.318^*^
Well	84 (23.9%)	96 (27.0%)		54 (23.4%)	60 (26.0%)	
Moderate	192 (54.5%)	175 (49.3%)		126 (54.5%)	110 (47.6%)	
Poor	76 (21.6%)	84 (23.7%)		51 (22.1%)	61 (26.4%)	
pT stage, n (%)			0.033^*^			0.955^*^
T1	60 (17.0%)	47 (13.2%)		35 (15.2%)	37 (16.0%)	
T2	107 (30.4%)	87 (24.5%)		56 (24.2%)	54 (23.4%)	
T3	185 (52.6%)	221 (62.3%)		140 (60.6%)	140 (60.6%)	
Surgical approaches, n (%)			< 0.001^*^			0.095^**^
Sweet	287 (81.5%)	194 (54.6%)		178 (77.1%)	179 (77.5%)	
Ivor-Lewis	15 (4.3%)	9 (2.5%)		10 (4.3%)	9 (3.9%)	
McKeown	37 (10.5%)	145 (40.8%)		33 (14.3%)	41 (17.7%)	
Other	13 (3.7%)	7 (2.1%)		10 (4.3%)	2 (0.9%)	
Transthoracic laterality, n (%)			< 0.001^**^			0.735^**^
Left	293 (83.2%)	196 (55.2%)		182 (78.8%)	179 (77.5%)	
Right	57 (16.2%)	156 (43.9%)		48 (20.8%)	52 (22.5%)	
Other	2 (0.6%)	3 (0.9%)		1 (0.4%)	0 (0.0%)	
Two-field dissection, n (%)			0.093^*^			1.000^*^
No	30 (8.5%)	44 (12.4%)		7 (3.0%)	8 (3.5%)	
Yes	322 (91.5%)	311 (97.6%)		224 (97.0%)	223 (96.5%)	
Three-field dissection, n (%)			< 0.001^**^			1.000^**^
No	349 (99.1%)	316 (89.0%)		228 (98.7%)	227 (98.3%)	
Yes	3 (0.9%)	39 (11.0%)		3 (1.3%)	4 (1.7%)	
Tumor location, n (%)			0.647^*^			0.776^*^
Upper	31 (8.8%)	36 (10.1%)		14 (6.1%)	12 (5.2%)	
Middle	123 (34.9%)	131 (36.9%)		76 (32.9%)	83 (35.9%)	
Lower	198 (56.3%)	188 (53.0%)		141 (61.0%)	136 (58.9%)	
Age (year), n (%)			0.312^*^			0.453^*^
≤ 60	196 (55.7%)	211 (59.4%)		134 (58.0%)	126 (54.5%)	
> 60	156 (44.3%)	144 (40.6%)		97 (42.0%)	105 (45.5%)	
Median (interquartile range)	59 (14)	59 (12)	0.972^***^	59 (15)	60 (12)	0.082^***^
Tumor length (cm)			0.013^**^			0.705^*^
≤ 3	192 (55.2%)	162 (45.8%)		107 (46.9%)	112 (48.7%)	
> 3	156 (44.8%)	192 (54.2%)		121 (53.1%)	118 (51.3%)	
Median (interquartile range)	3.0 (2.0)	3.5 (2.5)	0.001^***^	3.5 (2.0)	3.5 (2.5)	0.776^***^

*NELN* the number of examined lymph node; *: chi-squared test; **: Fisher’s exact test; ***: Mann-Whitney *U* test

There were 5 patients with missing data in tumor length

**Table 2:** Clinical characteristic of esophageal squamous cell carcinoma patients with stage T1–3N0M0 from SEER database

	All patients (*N* = 151)	NELNs		
		≤15 (*N* = 98)	> 15 (*N* = 53)	
Variables				*P* value
Sex, n (%)				0.853^*^
Male	87 (57.6%)	57 (58.2%)	30 (56.6%)	
Female	64 (42.4%)	41 (41.8%)	23 (43.4%)	
Race, n (%)				0.001^***^
White patients	104 (68.9%)	69 (70.4%)	35 (66.0%)	
Black patients	26 (17.2%)	22 (22.4%)	4 (7.6%)	
Other patients	21 (13.9%)	7 (7.2%)	14 (26.4%)	
Age (year), n (%)				0.155^*^
≤ 60	42 (27.8%)	31 (31.6%)	11 (20.8%)	
> 60	109 (72.2%)	67 (68.4%)	42 (79.2%)	
Tumor differentiation, n (%)				0.325^*^
Well	20 (13.2%)	10 (10.2%)	10 (18.9%)	
Moderate	82 (54.3%)	55 (56.1%)	27 (50.9%)	
Poor	49 (32.5%)	33 (33.7%)	16 (30.2%)	
pT stage, n (%)				0.126^*^
T1	61 (40.4%)	40 (40.8%)	21 (39.6%)	
T2	30 (19.9%)	15 (15.3%)	15 (28.3%)	
T3	60 (39.7%)	43 (43.9%)	17 (32.1%)	
Tumor location, n (%)				0.129^***^
Upper	15 (9.9%)	13 (13.3%)	2 (3.8%)	
Middle	75 (49.7%)	49 (50.0%)	26 (49.1%)	
Lower	61 (40.4%)	36 (36.7%)	25 (47.1%)	
Tumor length (cm)				
Median (interquartile range)	3.0 (2.6)	3.5 (2.5)	2.5 (2.5)	0.121^**^

*SEER* Surveillance Epidemiology and End Results, *NELN* the number of examined lymph node; *: chi-squared test; **: Mann-Whitney *U* test; ***: Fisher exact test

### Univariable and multivariable analyses

**Fig. 3:**
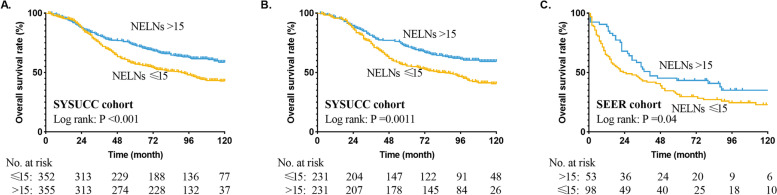
Overall survival curve for esophageal squamous cell cancer patients with stage T1–3N0M0 according to the number of examined lymph nodes in the unmatched cohort of Sun Yat-sen University Cancer Center (**A**), matched patients of Sun Yat-sen University Cancer Center (**B**), and Surveillance Epidemiology and End Results database (**C**)

As shown in Table 3, univariable and multivariable analyses identified NELNs as an independent significant overall survival prognostic factor adjusting for other factors in ESCC patients with stage T1–3N0M0 (adjusted HR 0.566, 95%CI 0.445–0.720; *p* < 0.001). Our results revealed that the 36-month and 60-month overall survival in the subgroup of NELNs > 15 vs. that in the subgroup of NELNs ≤15 was 77% vs. 65 and 69% vs. 55%, respectively in our cancer center. We found that there was a significant difference between the NELNs ≤15 and NELNs > 15 groups (Fig. 3A, unadjusted HR 0.630, 95% CI, 0.506–0.784, *p* < 0.001).

**Table 3:** Univariable and multivariable Cox regression analyses for prognostic factors in patients from Sun Yat-sen University Cancer Center cohort before propensity score matching

	Univariable analyses			Multivariable analyses		
	HR	95% CI	***P***-Value	HR	95% CI	***P***-Value
Sex						
Male	1	reference		1	reference	
Female	0.935	0.733–1.193	0.590	0.876	0.664–1.155	0.349
Age (years)						
≤ 60	1	reference		1	reference	
> 60	1.342	1.083–1.662	0.007	1.264	1.018–1.569	0.034
Drinking history						
No	1	reference		1	reference	
Yes	1.421	1.134–1.780	0.002	1.673	1.303–2.149	< 0.001
Tumor length (continuous)	1.007	0.941–1.078	0.832			
NELNs						
≤ 15	1	reference		1	reference	
> 15	0.630	0.506–0.784	< 0.001	0.566	0.445–0.720	< 0.001
Tumor differentiation						
Well	1	reference		1	reference	
Moderate	1.088	0.833–1.420	0.537	1.057	0.808–1.383	0.687
Poor	1.500	1.110–2.026	0.008	1.812	1.334–2.461	< 0.001
pT stage						
T1	1	reference		1	reference	
T2	1.132	0.776–1.652	0.520	1.236	0.840–1.819	0.283
T3	1.584	1.130–2.221	0.008	1.856	1.312–2.626	< 0.001
Tumor Location						
Upper	1	reference		1	reference	
Middle	0.960	0.665–1.386	0.828	0.899	0.605–1.305	0.547
Lower	0.659	0.460–0.946	0.024	0.491	0.331–0.729	< 0.001
Smoking history						
No	1	reference				
Yes	1.087	0.871–1.358	0.461			
Surgical approaches						
Sweet	1	reference		1	reference	
Ivor-Lewis	1.922	1.174–3.144	0.009	1.568	0.948–2.595	0.080
McKeown	0.782	0.602–1.017	0.066	0.749	0.543–1.033	0.078
Other	0.790	0.390–1.599	0.512	0.815	0.400–1.661	0.573
Transthoracic laterality						
Left	1	reference				
Right	0.856	0.673–1.088	0.204			
Other	0.879	0.218–3.536	0.855			
Two-field dissection						
No	1	reference		1	reference	
Yes	0.979	0.687–1.396	0.907	0.930	0.543–1.593	0.791
Three-field dissection						
No	1	reference		1	reference	
Yes	1.063	0.669–1.689	0.797	1.366	0.658–2.836	0.403

*HR* hazard ratio, *CI* confidence interval, *NELN* the number of examined lymph node; Cox regression’s method was Enter selection

Based on above results, we performed propensity score matching in the cohort from our cancer center, and got 231 pairs patients in the end. Chi-squared statistical test, and Mann-Whitney *U* test were used to estimate the quality the propensity score matching (Table 1**).** There was no significant difference in the other clinical indictors between group of NELNs ≤15 and group of NELNs > 15. In the matched cohort, our results revealed that the 36-month and 60-month overall survival in the subgroup of NELNs > 15 vs. that in the subgroup of NELNs ≤15 was 77% vs. 64 and 68% vs. 53%, respectively. Patients with NELNs > 15 might have survival benefit over patients of NELNs ≤15 (Fig. 3B).

### Validation for the survival effect of NELNs

**Fig. 4:**
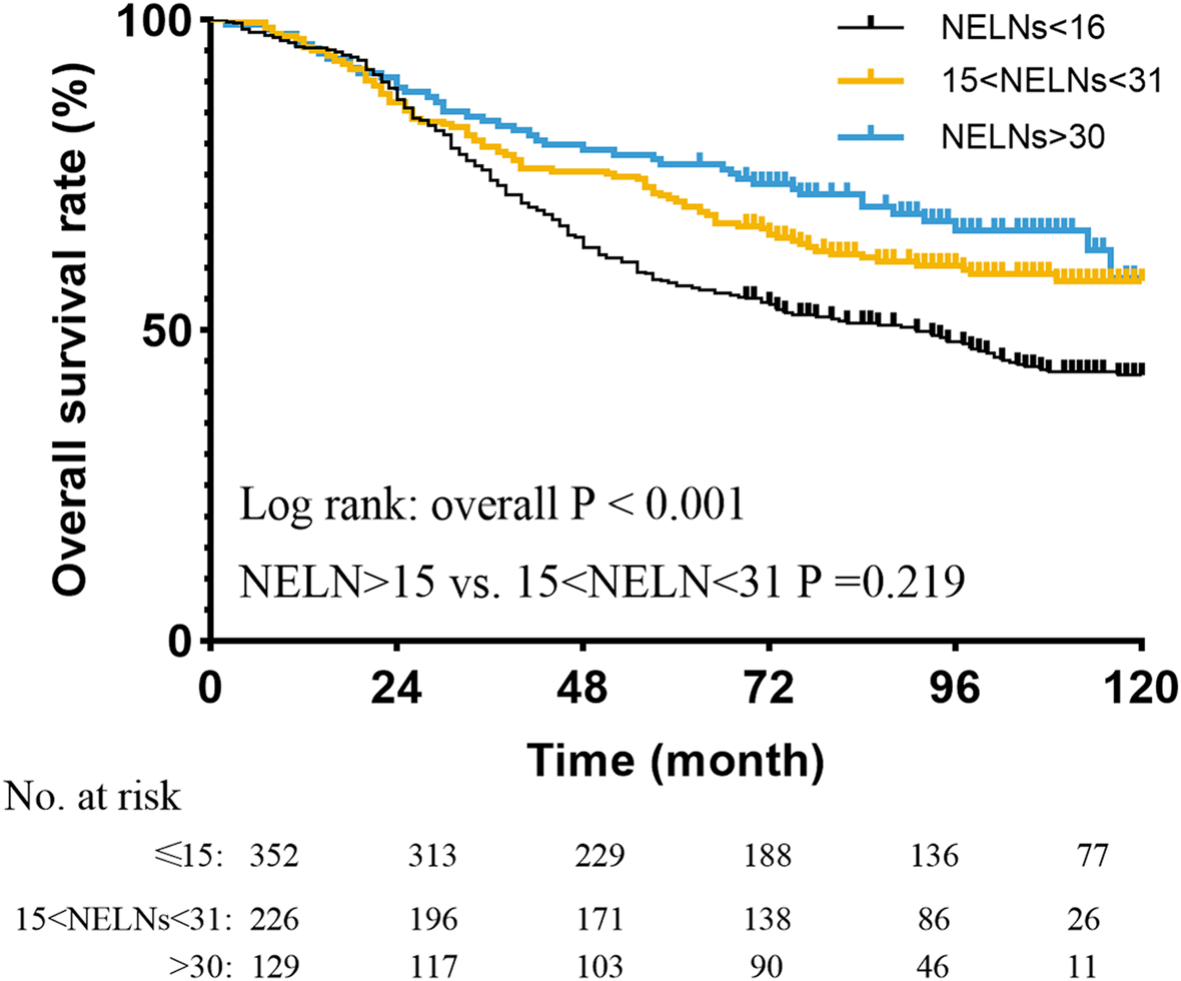
Overall survival curve for esophageal squamous cell cancer patients with stage T1–3N0M0 according to the number of examined lymph nodes

In order to validate the impact of NELNs on overall survival in pathological stage T1–3N0M0 ESCC patients, we collected 151 patients from SEER database as an external validation cohort. The same NELNs cutoff of 15 allowed us to stratify the patients within validation group into the subgroup of NELNs ≤15 with a significantly lower overall survival and the subgroup of NELNs > 15 with higher overall survival (Log rank: *p* = 0.044, Fig. 3C). Our results revealed that the 12-month, 36-month and 60-month overall survival in the subgroup of NELNs > 15 vs. that in the subgroup of NELNs ≤15 was 68% vs. 50, 45% vs. 41 and 43% vs. 29%, respectively in the SEER cohort. Besides, multivariable analysis also confirmed that NELNs > 15 could serve as a protective prognostic factor in those ESCC patients (adjusted HR 0.650, 95%CI 0.431–0.979, *p* = 0.039, Table 4). To further explore the effect of more NELNs on survival, another cutoff value of 30 was selected. We drew the survival curves to compare them. The results revealed that patients with NELNs> 30 did not have significantly better survival than patients with 15 < NELNs< 31 (Fig. 4) statistically. However, the survival curve of patients with NELNs > 30 is better than those with NELNs 15–30.

**Table 4:** Univariable and multivariable Cox proportional hazard regression analyses for prognostic factors in Surveillance Epidemiology and End Results cohort

	Univariable analysis			Multivariable analysis		
	HR	95% CI	*P*-Value	HR	95% CI	*P*-Value
Sex						
Male	1	reference		1	reference	
Female	0.843	0.578–1.228	0.374	0.798	0.544–1.171	0.798
Age (years)						
≤ 60	1	reference		1	reference	
> 60	0.872	0.581–1.310	0.510	0.909	0.590–1.399	0.664
Tumor length (continuous)	1.008	0.999–1.017	0.099			
NELNs						
≤ 15	1	reference		1	reference	
> 15	0.661	0.442–0.989	0.044	0.650	0.431–0.979	0.039
Tumor differentiation						
Well-moderate	1	reference		1	reference	
Poor	1.192	0.806–1.763	0.378	1.128	0.756–1.684	0.554
pT stage						
T1	1	reference		1	reference	
T2-T3	1.792	1.216–2.642	0.003	1.861	1.256–2.756	0.002
Tumor Location						
Upper-middle	1	reference				
Lower	0.973	0.662–1.429	0.888			
Race						
White patients	1	reference		1	reference	
Other patients	1.035	0.694–1.544	0.865	0.984	0.647–1.497	0.984

*NELNs* the number of examined lymph nodes; Cox regression’s method was Enter selection

### Stratified effect of NELNs on different T stages

**Fig. 5:**
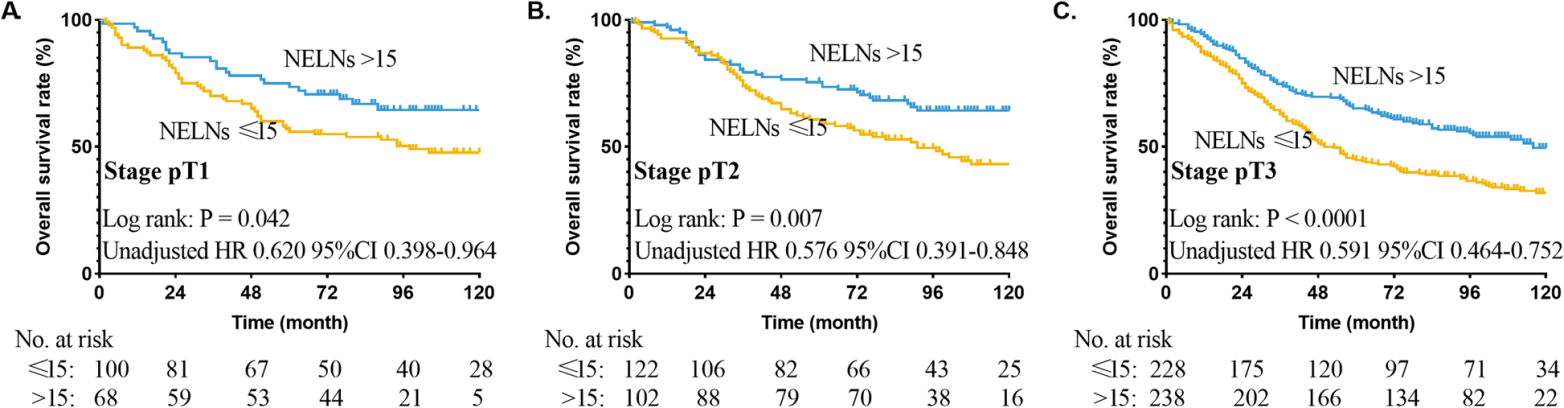
Overall survival curve for esophageal squamous cell cancer patients with stage T1–3N0M0 according to the number of examined lymph nodes in the cohort of pathological stage T1 (**A**), T2 (**B**) and T3 (**C**)

To further explore the stratified effect of NELNs on different T stages, we used the Kaplan-Meier analysis to draw survival curves. The results had shown that NELNs could identify the cohort with poor prognosis among patients with ESCC in different T stage (all *P* < 0.05, Fig. 5).

## Discussion

It is well known that ESCC is associated with poor prognosis and the five-year overall survival rate is about 20–40%. Previous studies suggested that certain factors had an effect on the prognosis of ESCC patients, however, the survival effect of NELNs on R0-resected ESCC patients with pathological stage T1–3N0M0 is still unclear. In the present study, we have analyzed the ESCC patients’ data from two cancer databases. Then, we have obtained an optimal cutoff value of NELNs as 15. Patients of NELNs ≤15 had a significantly lower overall survival than patients of NELNs > 15. Next, we have identified the NELNs as an independent prognostic factor adjusting for other confounders (such as sex, age, tumor differentiation, pT stage, and surgical approaches) by multivariable Cox regression. Another 151 patients from SEER database was used to validate the results based on data of Sun Yat-sen University Cancer Center. To further explore the effect of more NELNs on survival, another cutoff value of 30 was selected. We drew the survival curves to compare them. The results revealed that patients with NELNs> 30 did not have significantly better survival than patients with 15 < NELNs< 31 statistically. However, the survival curve of patients with NELNs > 30 is better than those with NELNs 15–30. The reason for the statistically insignificant results might be the number of patients with NELNs > 30 is too small, therefore, the survival curve shows a stratified trend, but the statistical difference is not significant enough. Besides, NELNs could identify the cohort with poor prognosis among patients with ESCC in different T stage. In terms of the clinical application, the indicator can be easily assessed. Information regarding NELNs can be obtained from postoperative pathology reports. Clinicians could use the information of NELNs to evaluate the prognosis of ESCC patients with pathological stage T1–3N0M0 after surgery, and give patients appropriate advice of follow-up strategy and adjuvant therapy.

In fact, previous studies have demonstrated that the number of lymph nodes removed during the surgery impact the survival outcomes on patients of esophageal cancer [8–11]. Those patients may gain survival advantages from more lymph nodes removed. Similarly, our study also has confirmed their findings. Therefore, surgeons are advised to dissect as many lymph nodes as they can during the operation. The majority of pathological subtype included in the study from *Nabi Rizk* et al was adenocarcinoma (80.7%), and the sample size was small (*N* = 65) [9]. However, the cases enrolled in our study all belong to squamous cell carcinoma, and the sample is large (Sun Yat-sen University Cancer Center, *N* = 707; SEER, *N* = 151). In addition, an international multicenter study from *Christian G. Peyre* et al enrolled 1181 patients with pathological stage T1–3N0M0 esophageal cancer, however, only less than 76 patients were from Asian [8]. Our study recruited 707 ESCC patients all from Asian and 151 patients from the United States. We suggested that this study might provide more prognostic information for Asian patients. At the end of the day, above researches all believe that the number of removed lymph nodes had effect on prognosis of esophageal cancer patients, and recommended surgeons performing dissection of more lymph nodes in the surgical resection.

Previous studies based on SEER database evaluated the association between lymph nodes count and prognosis in different malignant tumor, such as non-small cell lung cancer, male breast cancer, and colon cancer [16, 20–23]. Therefore, it’s feasible to use SEER data to confirm that lymph nodes count could affect the prognosis of ESCC with pathological stage T1–3N0M0. Of note, there still were some differences between SEER and Sun Yat-sen University Cancer Center database. First, the median of NELNs was bigger in our cancer center than that in the SEER database. Second, the majority pathological stage was T3 in our cancer center, however, the main part of ESCC belonged to stage T1 in the SEER database. Third, the distribution of age was quite different between two databases. Fourth, those two databases had very varied race/ ethics proportion. Thus, we have performed the propensity score matching in the database of our cancer center to reduce the selection bias and improve the reliability of our results before validation in SEER database. Based on this, we think that the results of this study are valuable and have some external application ability.

Recently, some clinical trials confirmed that many patients with locally advanced and resected ESCC could benefit from neoadjuvant therapy followed by surgery [24, 25]. The approach of neoadjuvant therapy included pembrolizumab combined with chemoradiotherapy, and chemoradiotherapy. In the study by *Yang Hong* et al, the patients considered as the diseases of classification T1-4N1M0/T4N0M0 entered in the trial [24]. And, in the clinical trial by *Li Chengqiang* et al, the diseases of this cohort belonged to the classification T3-4N0-2M0 [25]. Therefore, the evidence of the previous studies only suggested that local advanced patients might get survival benefit. However, as for relative early diseases, such as T1–3N0M0, there was not still enough evidence to support the benefit from neoadjuvant therapy. Thus, it is unclear whether such patients need to receive neoadjuvant therapy. Therefore, our results only uncover that the NELNs may be useful in choosing ESCC patients of stage T1–3N0M0 with surgery only for receiving adjuvant therapy. Our results could not apply in patients with neoadjuvant therapy. It is limitation of this study, thus, there is still a need for clinical trials to unravel the confusion.

### Limitations

However, there are some limitations in the study presented here. First of all, the sample size of ESCC patients was not large enough; the T stage was restricted to only the T1–3, and the data distribution of the T stage was not balanced. For further work, to improve this aspect, the sample size would need to be expended. Next, these findings could only provide certain reference information of prognosis to the clinicians but not the adjuvant treatment recommendations. The doctors would need to make decisions on the patients’ adjuvant treatment according to the relevant guidelines and clinical experience. Besides, the data of recurrence was not detailed, therefore, we could not explore the significance of NELN in the progression survival, and we also could not show the situation of the occult lymph node metastasis. In addition, the status of resected margin is not clear in the SEER database. According to the radiotherapy situation, we excluded the patients underwent adjuvant radiotherapy in the SEER database. To some extent, this method could ensure that all enrolled patients are R0-resected. Given the natural defects in the SEER database, more research is needed to provide detailed pathologic information to validate our findings.

## Conclusions

In conclusion, we have demonstrated that the number of examined lymph nodes is a significant prognostic factor in ESCC patients with pathological stage T1–3N0M0, and more than 15 examined lymph nodes is associated with better overall survival. Although the difference is not significant, the survival curve of patients with examined lymph nodes > 30 is better than those with examined lymph nodes 15–30. And the more examined lymph nodes cause the lesser occult lymph nodes metastasis and lead to better prognosis. To make patients require better survival outcomes, surgeons may need to dissect more lymph nodes based on actual situation during operation. Besides, pathologists should try to examine as many lymph nodes as possible to evaluate the pathological stage preciously. We believe that the number of examined lymph nodes can provide prognostic guidance for R0-resected ESCC patients with pathological stage T1–3N0M0, however, we need more validation from other studies.

## Data Availability

Any researches interested in this study could contact us for requiring the data, and our clinical data was uploaded in the Research Data Deposit (http://www.researchdata.org.cn/).

## Abbreviations

NELNs: The number of examined lymph nodes
ESCC: Esophageal squamous cell carcinoma
SEER: Surveillance Epidemiology and End Results
HR: Hazard ratio
CI: Confidence interval
pT: Pathological tumor
